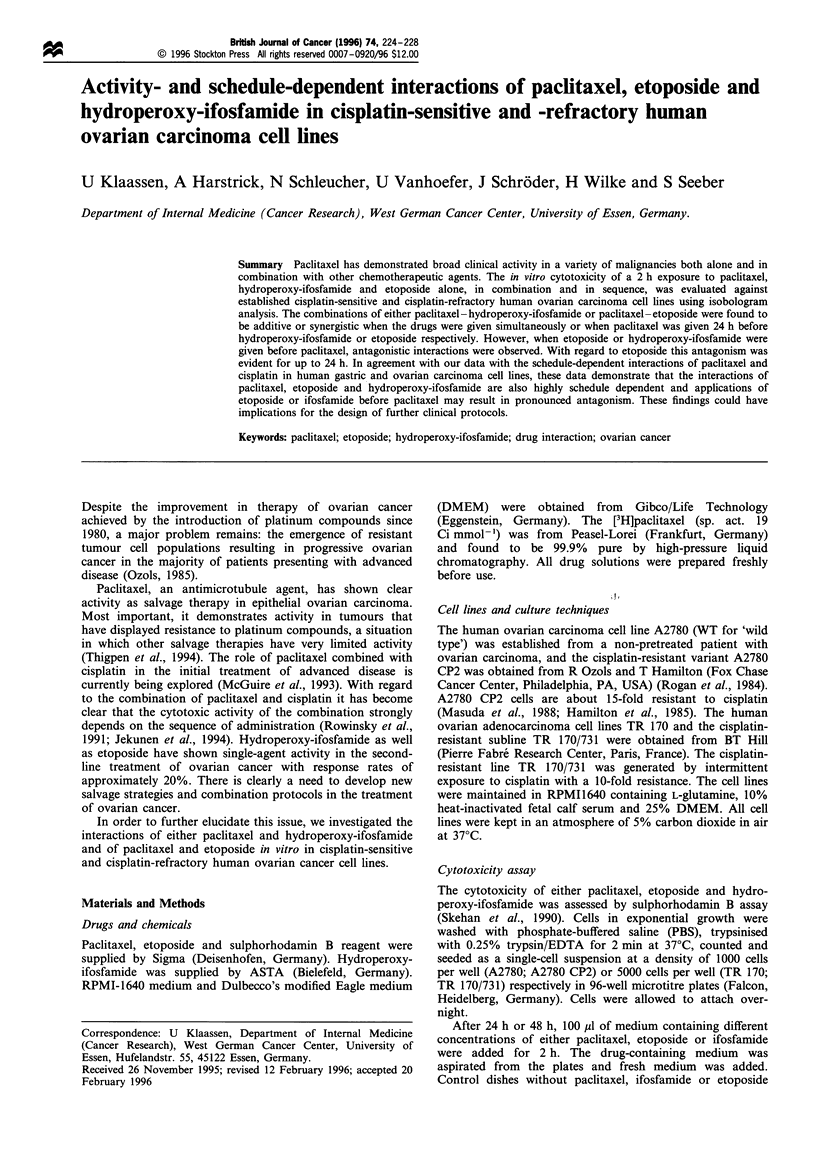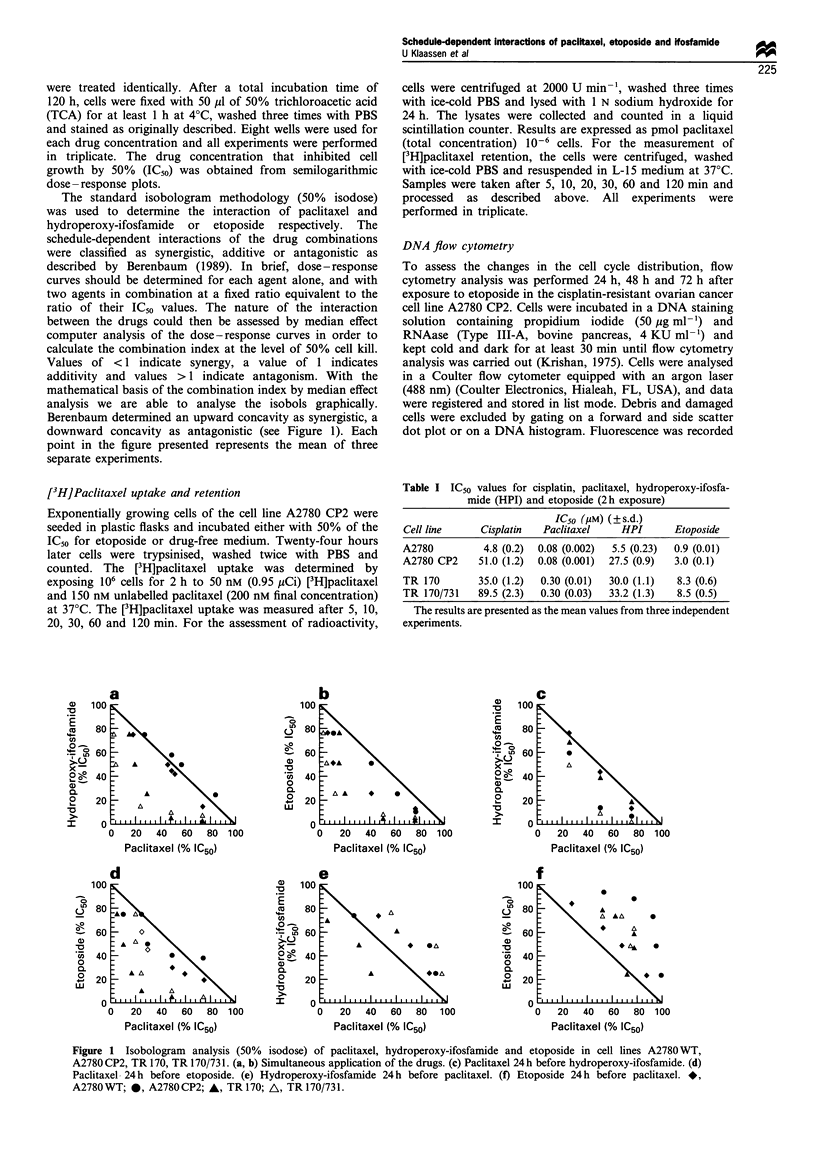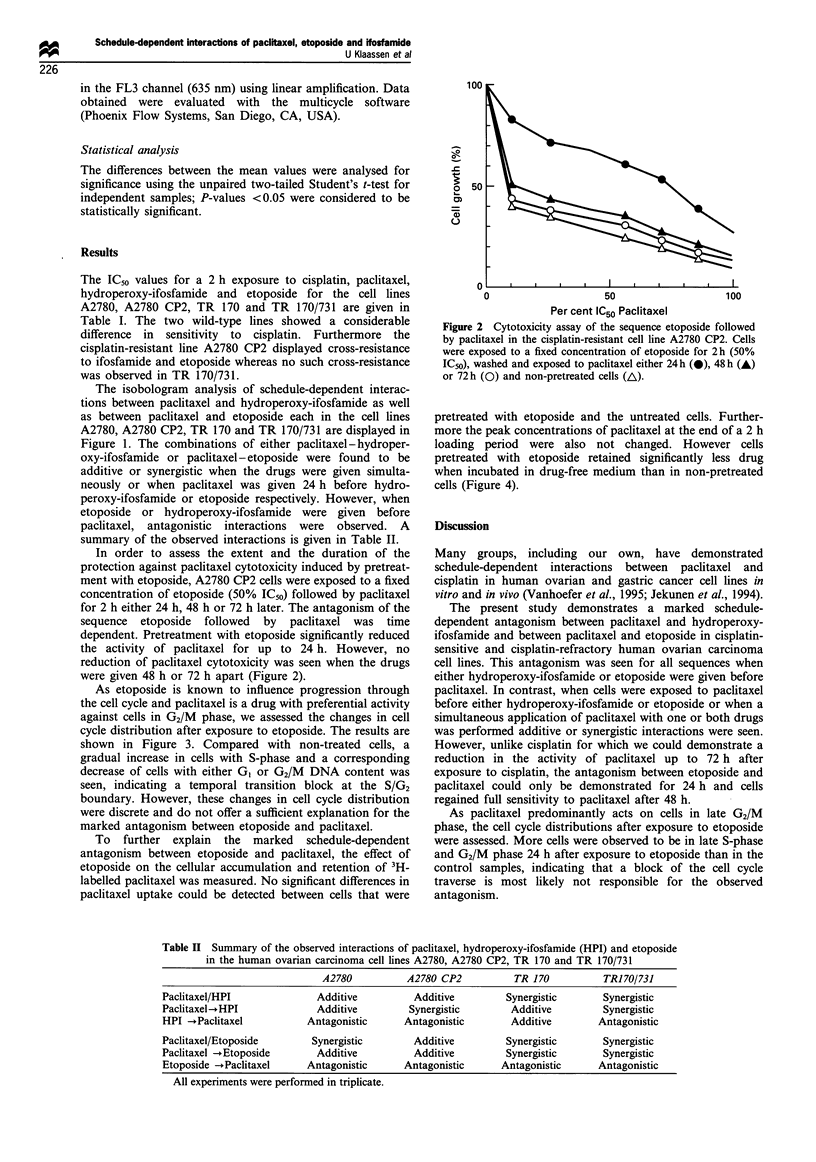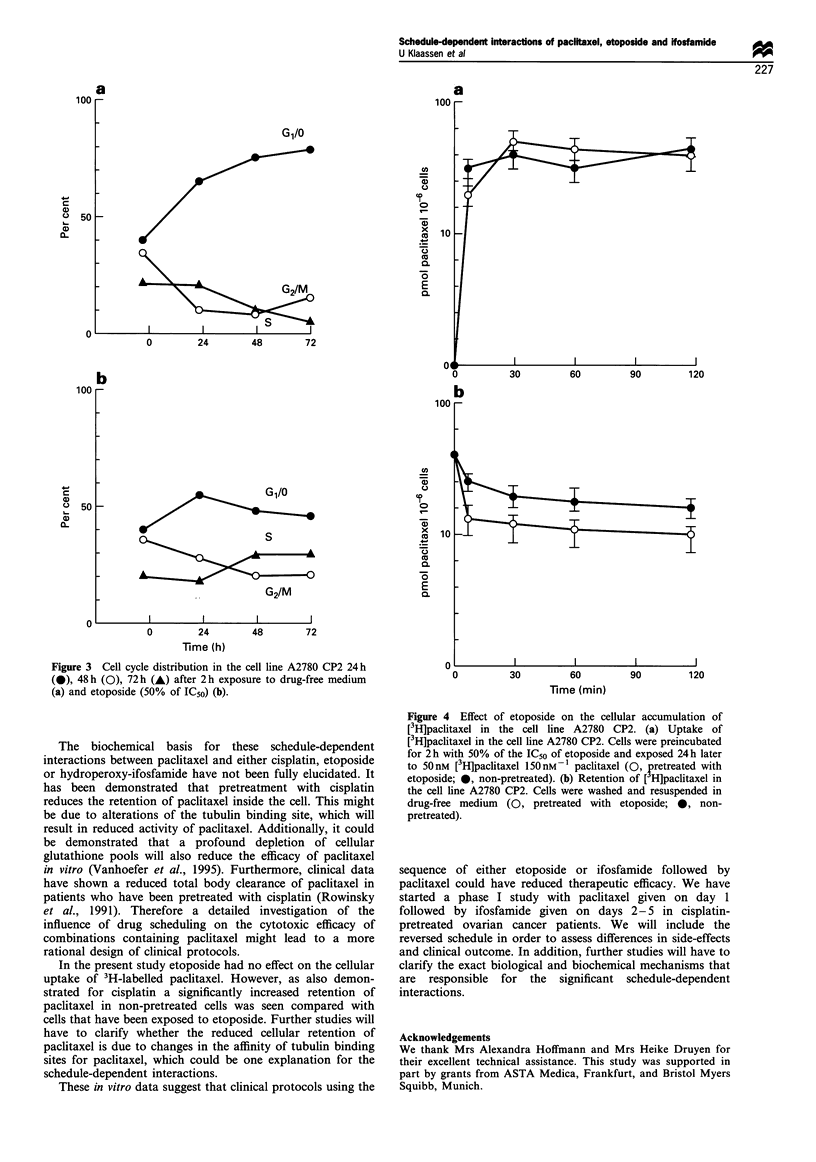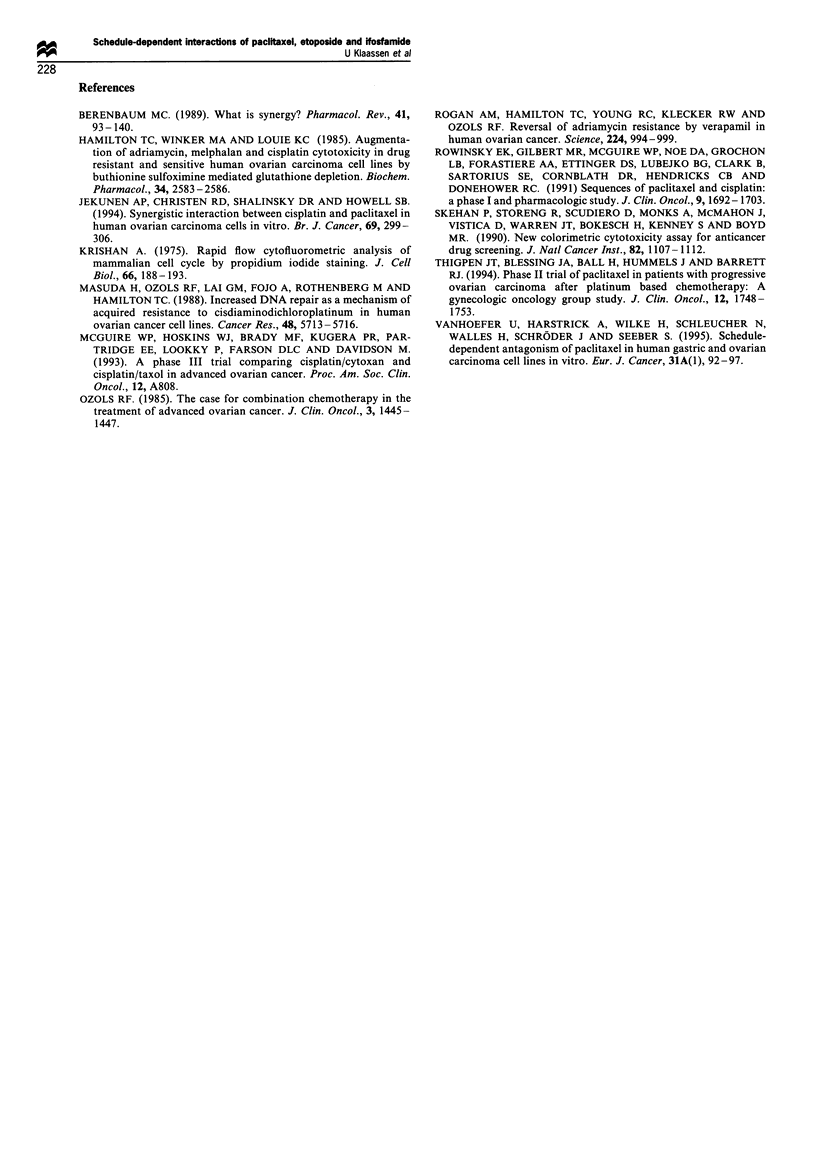# Activity- and schedule-dependent interactions of paclitaxel, etoposide and hydroperoxy-ifosfamide in cisplatin-sensitive and -refractory human ovarian carcinoma cell lines.

**DOI:** 10.1038/bjc.1996.341

**Published:** 1996-07

**Authors:** U. Klaassen, A. Harstrick, N. Schleucher, U. Vanhoefer, J. Schröder, H. Wilke, S. Seeber

**Affiliations:** Department of Internal Medicine (Cancer Research), West German Cancer Center, University of Essen, Germany.

## Abstract

Paclitaxel has demonstrated broad clinical activity in a variety of malignancies both alone and in combination with other chemotherapeutic agents. The in vitro cytotoxicity of a 2 h exposure to paclitaxel, hydroperoxy-ifosfamide and etoposide alone, in combination and in sequence, was evaluated against established cisplatin-sensitive and cisplatin-refractory human ovarian carcinoma cell lines using isobologram analysis. The combinations of either paclitaxel-hydroperoxy-ifosfamide or paclitaxel-etoposide were found to be additive or synergistic when the drugs were given simultaneously or when paclitaxel was given 24 h before hydroperoxy-ifosfamide or etoposide respectively. However, when etoposide or hydroperoxy-ifosfamide were given before paclitaxel, antagonistic interactions were observed. With regard to etoposide this antagonism was evident for up to 24 h. In agreement with our data with the schedule-dependent interactions of paclitaxel and cisplatin in human gastric and ovarian carcinoma cell lines, these data demonstrate that the interactions of paclitaxel, etoposide and hydroperoxy-ifosfamide are also highly schedule dependent and applications of etoposide or ifosfamide before paclitaxel may result in pronounced antagonism. These findings could have implications for the design of further clinical protocols.


British Journal of Cancer (1996) 74, 224-228
© 1996 Stockton Press All rights reserved 0007-0920/96 $12.00

### Activity- and schedule-dependent interactions of paclitaxel, etoposide and hydroperoxy-ifosfamide in cisplatin-sensitive and -refractory human ovarian carcinoma cell lines

U Klaassen, A Harstrick, N Schleucher, U Vanhoefer, J Schröder, H Wilke and S Seeber

*Department of Internal Medicine (Cancer Research), West German Cancer Center, University of Essen, Germany.*

**Summary** Paclitaxel has demonstrated broad clinical activity in a variety of malignancies both alone and in combination with other chemotherapeutic agents. The *in vitro* cytotoxicity of a 2 h exposure to paclitaxel, hydroperoxy-ifosfamide and etoposide alone, in combination and in sequence, was evaluated against established cisplatin-sensitive and cisplatin-refractory human ovarian carcinoma cell lines using isobologram analysis. The combinations of either paclitaxel–hydroperoxy-ifosfamide or paclitaxel–etoposide were found to be additive or synergistic when the drugs were given simultaneously or when paclitaxel was given 24 h before hydroperoxy-ifosfamide or etoposide respectively. However, when etoposide or hydroperoxy-ifosfamide were given before paclitaxel, antagonistic interactions were observed. With regard to etoposide this antagonism was evident for up to 24 h. In agreement with our data with the schedule-dependent interactions of paclitaxel and cisplatin in human gastric and ovarian carcinoma cell lines, these data demonstrate that the interactions of paclitaxel, etoposide and hydroperoxy-ifosfamide are also highly schedule dependent and applications of etoposide or ifosfamide before paclitaxel may result in pronounced antagonism. These findings could have implications for the design of further clinical protocols.

**Keywords:** paclitaxel; etoposide; hydroperoxy-ifosfamide; drug interaction; ovarian cancer

Despite the improvement in therapy of ovarian cancer achieved by the introduction of platinum compounds since 1980, a major problem remains: the emergence of resistant tumour cell populations resulting in progressive ovarian cancer in the majority of patients presenting with advanced disease (Ozols, 1985).

Paclitaxel, an antimicrotubule agent, has shown clear activity as salvage therapy in epithelial ovarian carcinoma. Most important, it demonstrates activity in tumours that have displayed resistance to platinum compounds, a situation in which other salvage therapies have very limited activity (Thigpen *et al.*, 1994). The role of paclitaxel combined with cisplatin in the initial treatment of advanced disease is currently being explored (McGuire *et al.*, 1993). With regard to the combination of paclitaxel and cisplatin it has become clear that the cytotoxic activity of the combination strongly depends on the sequence of administration (Rowinsky *et al.*, 1991; Jekunen *et al.*, 1994). Hydroperoxy-ifosfamide as well as etoposide have shown single-agent activity in the second-line treatment of ovarian cancer with response rates of approximately 20%. There is clearly a need to develop new salvage strategies and combination protocols in the treatment of ovarian cancer.

In order to further elucidate this issue, we investigated the interactions of either paclitaxel and hydroperoxy-ifosfamide and of paclitaxel and etoposide *in vitro* in cisplatin-sensitive and cisplatin-refractory human ovarian cancer cell lines.

### Materials and Methods

### *Drugs and chemicals*

Paclitaxel, etoposide and sulphorhodamin B reagent were supplied by Sigma (Deisenhofen, Germany). Hydroperoxy-ifosfamide was supplied by ASTA (Bielefeld, Germany). RPMI-1640 medium and Dulbecco's modified Eagle medium (DMEM) were obtained from Gibco/Life Technology (Eggenstein, Germany). The [$^3$H]paclitaxel (sp. act. 19 Ci mmol$^{-1}$) was from Peasel-Lorei (Frankfurt, Germany) and found to be 99.9% pure by high-pressure liquid chromatography. All drug solutions were prepared freshly before use.

### *Cell lines and culture techniques*

The human ovarian carcinoma cell line A2780 (WT for 'wild type') was established from a non-pretreated patient with ovarian carcinoma, and the cisplatin-resistant variant A2780 CP2 was obtained from R Ozols and T Hamilton (Fox Chase Cancer Center, Philadelphia, PA, USA) (Rogan *et al.*, 1984). A2780 CP2 cells are about 15-fold resistant to cisplatin (Masuda *et al.*, 1988; Hamilton *et al.*, 1985). The human ovarian adenocarcinoma cell lines TR 170 and the cisplatin-resistant subline TR 170/731 were obtained from BT Hill (Pierre Fabré Research Center, Paris, France). The cisplatin-resistant line TR 170/731 was generated by intermittent exposure to cisplatin with a 10-fold resistance. The cell lines were maintained in RPMI1640 containing L-glutamine, 10% heat-inactivated fetal calf serum and 25% DMEM. All cell lines were kept in an atmosphere of 5% carbon dioxide in air at 37°C.

### *Cytotoxicity assay*

The cytotoxicity of either paclitaxel, etoposide and hydroperoxy-ifosfamide was assessed by sulphorhodamin B assay (Skehan *et al.*, 1990). Cells in exponential growth were washed with phosphate-buffered saline (PBS), trypsinised with 0.25% trypsin/EDTA for 2 min at 37°C, counted and seeded as a single-cell suspension at a density of 1000 cells per well (A2780; A2780 CP2) or 5000 cells per well (TR 170; TR 170/731) respectively in 96-well microtitre plates (Falcon, Heidelberg, Germany). Cells were allowed to attach overnight.

After 24 h or 48 h, 100 μl of medium containing different concentrations of either paclitaxel, etoposide or ifosfamide were added for 2 h. The drug-containing medium was aspirated from the plates and fresh medium was added. Control dishes without paclitaxel, ifosfamide or etoposide

Correspondence: U Klaassen, Department of Internal Medicine (Cancer Research), West German Cancer Center, University of Essen, Hufelandstr. 55, 45122 Essen, Germany.
Received 26 November 1995; revised 12 February 1996; accepted 20 February 1996

were treated identically. After a total incubation time of 120 h, cells were fixed with 50 $\mu$l of 50% trichloroacetic acid (TCA) for at least 1 h at 4°C, washed three times with PBS and stained as originally described. Eight wells were used for each drug concentration and all experiments were performed in triplicate. The drug concentration that inhibited cell growth by 50% ($IC_{50}$) was obtained from semilogarithmic dose–response plots.

The standard isobologram methodology (50% isodose) was used to determine the interaction of paclitaxel and hydroperoxy-ifosfamide or etoposide respectively. The schedule-dependent interactions of the drug combinations were classified as synergistic, additive or antagonistic as described by Berenbaum (1989). In brief, dose–response curves should be determined for each agent alone, and with two agents in combination at a fixed ratio equivalent to the ratio of their $IC_{50}$ values. The nature of the interaction between the drugs could then be assessed by median effect computer analysis of the dose–response curves in order to calculate the combination index at the level of 50% cell kill. Values of $<1$ indicate synergy, a value of 1 indicates additivity and values $>1$ indicate antagonism. With the mathematical basis of the combination index by median effect analysis we are able to analyse the isobols graphically. Berenbaum determined an upward concavity as synergistic, a downward concavity as antagonistic (see Figure 1). Each point in the figure presented represents the mean of three separate experiments.

### *[$^3$H]Paclitaxel uptake and retention*

Exponentially growing cells of the cell line A2780 CP2 were seeded in plastic flasks and incubated either with 50% of the $IC_{50}$ for etoposide or drug-free medium. Twenty-four hours later cells were trypsinised, washed twice with PBS and counted. The [$^3$H]paclitaxel uptake was determined by exposing $10^6$ cells for 2 h to 50 nM (0.95 $\mu$Ci) [$^3$H]paclitaxel and 150 nM unlabelled paclitaxel (200 nM final concentration) at 37°C. The [$^3$H]paclitaxel uptake was measured after 5, 10, 20, 30, 60 and 120 min. For the assessment of radioactivity, cells were centrifuged at 2000 U $min^{-1}$, washed three times with ice-cold PBS and lysed with 1 N sodium hydroxide for 24 h. The lysates were collected and counted in a liquid scintillation counter. Results are expressed as pmol paclitaxel (total concentration) $10^{-6}$ cells. For the measurement of [$^3$H]paclitaxel retention, the cells were centrifuged, washed with ice-cold PBS and resuspended in L-15 medium at 37°C. Samples were taken after 5, 10, 20, 30, 60 and 120 min and processed as described above. All experiments were performed in triplicate.

### *DNA flow cytometry*

To assess the changes in the cell cycle distribution, flow cytometry analysis was performed 24 h, 48 h and 72 h after exposure to etoposide in the cisplatin-resistant ovarian cancer cell line A2780 CP2. Cells were incubated in a DNA staining solution containing propidium iodide (50 $\mu g\ ml^{-1}$) and RNAase (Type III-A, bovine pancreas, 4 KU $ml^{-1}$) and kept cold and dark for at least 30 min until flow cytometry analysis was carried out (Krishan, 1975). Cells were analysed in a Coulter flow cytometer equipped with an argon laser (488 nm) (Coulter Electronics, Hialeah, FL, USA), and data were registered and stored in list mode. Debris and damaged cells were excluded by gating on a forward and side scatter dot plot or on a DNA histogram. Fluorescence was recorded

**Table I** $IC_{50}$ values for cisplatin, paclitaxel, hydroperoxy-ifosfamide (HPI) and etoposide (2 h exposure)

| | $IC_{50}$ ($\mu$M) ($\pm$s.d.) | | | |
|---|---|---|---|---|
| *Cell line* | *Cisplatin* | *Paclitaxel* | *HPI* | *Etoposide* |
| A2780 | 4.8 (0.2) | 0.08 (0.002) | 5.5 (0.23) | 0.9 (0.01) |
| A2780 CP2 | 51.0 (1.2) | 0.08 (0.001) | 27.5 (0.9) | 3.0 (0.1) |
| TR 170 | 35.0 (1.2) | 0.30 (0.01) | 30.0 (1.1) | 8.3 (0.6) |
| TR 170/731 | 89.5 (2.3) | 0.30 (0.03) | 33.2 (1.3) | 8.5 (0.5) |

The results are presented as the mean values from three independent experiments.

**Figure 1** Isobologram analysis (50% isodose) of paclitaxel, hydroperoxy-ifosfamide and etoposide in cell lines A2780WT, A2780CP2, TR170, TR170/731. (**a**, **b**) Simultaneous application of the drugs. (**c**) Paclitaxel 24 h before hydroperoxy-ifosfamide. (**d**) Paclitaxel 24 h before etoposide. (**e**) Hydroperoxy-ifosfamide 24 h before paclitaxel. (**f**) Etoposide 24 h before paclitaxel. ◆, A2780WT; ●, A2780CP2; ▲, TR170; △, TR170/731.

in the FL3 channel (635 nm) using linear amplification. Data obtained were evaluated with the multicycle software (Phoenix Flow Systems, San Diego, CA, USA).

*Statistical analysis*

The differences between the mean values were analysed for significance using the unpaired two-tailed Student's *t*-test for independent samples; *P*-values $<0.05$ were considered to be statistically significant.

### Results

The $IC_{50}$ values for a 2 h exposure to cisplatin, paclitaxel, hydroperoxy-ifosfamide and etoposide for the cell lines A2780, A2780 CP2, TR 170 and TR 170/731 are given in Table I. The two wild-type lines showed a considerable difference in sensitivity to cisplatin. Furthermore the cisplatin-resistant line A2780 CP2 displayed cross-resistance to ifosfamide and etoposide whereas no such cross-resistance was observed in TR 170/731.

The isobologram analysis of schedule-dependent interactions between paclitaxel and hydroperoxy-ifosfamide as well as between paclitaxel and etoposide each in the cell lines A2780, A2780 CP2, TR 170 and TR 170/731 are displayed in Figure 1. The combinations of either paclitaxel–hydroperoxy-ifosfamide or paclitaxel–etoposide were found to be additive or synergistic when the drugs were given simultaneously or when paclitaxel was given 24 h before hydroperoxy-ifosfamide or etoposide respectively. However, when etoposide or hydroperoxy-ifosfamide were given before paclitaxel, antagonistic interactions were observed. A summary of the observed interactions is given in Table II.

In order to assess the extent and the duration of the protection against paclitaxel cytotoxicity induced by pretreatment with etoposide, A2780 CP2 cells were exposed to a fixed concentration of etoposide (50% $IC_{50}$) followed by paclitaxel for 2 h either 24 h, 48 h or 72 h later. The antagonism of the sequence etoposide followed by paclitaxel was time dependent. Pretreatment with etoposide significantly reduced the activity of paclitaxel for up to 24 h. However, no reduction of paclitaxel cytotoxicity was seen when the drugs were given 48 h or 72 h apart (Figure 2).

As etoposide is known to influence progression through the cell cycle and paclitaxel is a drug with preferential activity against cells in $G_2$/M phase, we assessed the changes in cell cycle distribution after exposure to etoposide. The results are shown in Figure 3. Compared with non-treated cells, a gradual increase in cells with S-phase and a corresponding decrease of cells with either $G_1$ or $G_2$/M DNA content was seen, indicating a temporal transition block at the S/$G_2$ boundary. However, these changes in cell cycle distribution were discrete and do not offer a sufficient explanation for the marked antagonism between etoposide and paclitaxel.

To further explain the marked schedule-dependent antagonism between etoposide and paclitaxel, the effect of etoposide on the cellular accumulation and retention of $^3$H-labelled paclitaxel was measured. No significant differences in paclitaxel uptake could be detected between cells that were pretreated with etoposide and the untreated cells. Furthermore the peak concentrations of paclitaxel at the end of a 2 h loading period were also not changed. However cells pretreated with etoposide retained significantly less drug when incubated in drug-free medium than in non-pretreated cells (Figure 4).

**Figure 2** Cytotoxicity assay of the sequence etoposide followed by paclitaxel in the cisplatin-resistant cell line A2780 CP2. Cells were exposed to a fixed concentration of etoposide for 2 h (50% $IC_{50}$), washed and exposed to paclitaxel either 24 h (●), 48 h (▲) or 72 h (○) and non-pretreated cells (△).

### Discussion

Many groups, including our own, have demonstrated schedule-dependent interactions between paclitaxel and cisplatin in human ovarian and gastric cancer cell lines *in vitro* and *in vivo* (Vanhoefer *et al.*, 1995; Jekunen *et al.*, 1994).

The present study demonstrates a marked schedule-dependent antagonism between paclitaxel and hydroperoxy-ifosfamide and between paclitaxel and etoposide in cisplatin-sensitive and cisplatin-refractory human ovarian carcinoma cell lines. This antagonism was seen for all sequences when either hydroperoxy-ifosfamide or etoposide were given before paclitaxel. In contrast, when cells were exposed to paclitaxel before either hydroperoxy-ifosfamide or etoposide or when a simultaneous application of paclitaxel with one or both drugs was performed additive or synergistic interactions were seen. However, unlike cisplatin for which we could demonstrate a reduction in the activity of paclitaxel up to 72 h after exposure to cisplatin, the antagonism between etoposide and paclitaxel could only be demonstrated for 24 h and cells regained full sensitivity to paclitaxel after 48 h.

As paclitaxel predominantly acts on cells in late $G_2$/M phase, the cell cycle distributions after exposure to etoposide were assessed. More cells were observed to be in late S-phase and $G_2$/M phase 24 h after exposure to etoposide than in the control samples, indicating that a block of the cell cycle traverse is most likely not responsible for the observed antagonism.

**Table II** Summary of the observed interactions of paclitaxel, hydroperoxy-ifosfamide (HPI) and etoposide in the human ovarian carcinoma cell lines A2780, A2780 CP2, TR 170 and TR 170/731

| | *A2780* | *A2780 CP2* | *TR 170* | *TR170/731* |
|---|---|---|---|---|
| Paclitaxel/HPI | Additive | Additive | Synergistic | Synergistic |
| Paclitaxel→HPI | Additive | Synergistic | Additive | Synergistic |
| HPI →Paclitaxel | Antagonistic | Antagonistic | Additive | Antagonistic |
| Paclitaxel/Etoposide | Synergistic | Additive | Synergistic | Synergistic |
| Paclitaxel →Etoposide | Additive | Additive | Synergistic | Synergistic |
| Etoposide →Paclitaxel | Antagonistic | Antagonistic | Antagonistic | Antagonistic |

All experiments were performed in triplicate.

**Figure 3** Cell cycle distribution in the cell line A2780 CP2 24 h (●), 48 h (○), 72 h (▲) after 2 h exposure to drug-free medium (**a**) and etoposide (50% of $IC_{50}$) (**b**).

**Figure 4** Effect of etoposide on the cellular accumulation of [$^3$H]paclitaxel in the cell line A2780 CP2. (**a**) Uptake of [$^3$H]paclitaxel in the cell line A2780 CP2. Cells were preincubated for 2 h with 50% of the $IC_{50}$ of etoposide and exposed 24 h later to 50 nM [$^3$H]paclitaxel 150 nM$^{-1}$ paclitaxel (○, pretreated with etoposide; ●, non-pretreated). (**b**) Retention of [$^3$H]paclitaxel in the cell line A2780 CP2. Cells were washed and resuspended in drug-free medium (○, pretreated with etoposide; ●, non-pretreated).

The biochemical basis for these schedule-dependent interactions between paclitaxel and either cisplatin, etoposide or hydroperoxy-ifosfamide have not been fully elucidated. It has been demonstrated that pretreatment with cisplatin reduces the retention of paclitaxel inside the cell. This might be due to alterations of the tubulin binding site, which will result in reduced activity of paclitaxel. Additionally, it could be demonstrated that a profound depletion of cellular glutathione pools will also reduce the efficacy of paclitaxel *in vitro* (Vanhoefer *et al.*, 1995). Furthermore, clinical data have shown a reduced total body clearance of paclitaxel in patients who have been pretreated with cisplatin (Rowinsky *et al.*, 1991). Therefore a detailed investigation of the influence of drug scheduling on the cytotoxic efficacy of combinations containing paclitaxel might lead to a more rational design of clinical protocols.

In the present study etoposide had no effect on the cellular uptake of $^3$H-labelled paclitaxel. However, as also demonstrated for cisplatin a significantly increased retention of paclitaxel in non-pretreated cells was seen compared with cells that have been exposed to etoposide. Further studies will have to clarify whether the reduced cellular retention of paclitaxel is due to changes in the affinity of tubulin binding sites for paclitaxel, which could be one explanation for the schedule-dependent interactions.

These *in vitro* data suggest that clinical protocols using the sequence of either etoposide or ifosfamide followed by paclitaxel could have reduced therapeutic efficacy. We have started a phase I study with paclitaxel given on day 1 followed by ifosfamide given on days 2–5 in cisplatin-pretreated ovarian cancer patients. We will include the reversed schedule in order to assess differences in side-effects and clinical outcome. In addition, further studies will have to clarify the exact biological and biochemical mechanisms that are responsible for the significant schedule-dependent interactions.

**Acknowledgements**

We thank Mrs Alexandra Hoffmann and Mrs Heike Druyen for their excellent technical assistance. This study was supported in part by grants from ASTA Medica, Frankfurt, and Bristol Myers Squibb, Munich.

### References

BERENBAUM MC. (1989). What is synergy? *Pharmacol. Rev.*, **41**, 93-140.

HAMILTON TC, WINKER MA AND LOUIE KC (1985). Augmentation of adriamycin, melphalan and cisplatin cytotoxicity in drug resistant and sensitive human ovarian carcinoma cell lines by buthionine sulfoximine mediated glutathione depletion. *Biochem. Pharmacol.*, **34**, 2583-2586.

JEKUNEN AP, CHRISTEN RD, SHALINSKY DR AND HOWELL SB. (1994). Synergistic interaction between cisplatin and paclitaxel in human ovarian carcinoma cells in vitro. *Br. J. Cancer*, **69**, 299-306.

KRISHAN A. (1975). Rapid flow cytofluorometric analysis of mammalian cell cycle by propidium iodide staining. *J. Cell Biol.*, **66**, 188-193.

MASUDA H, OZOLS RF, LAI GM, FOJO A, ROTHENBERG M AND HAMILTON TC. (1988). Increased DNA repair as a mechanism of acquired resistance to cisdiaminodichloroplatinum in human ovarian cancer cell lines. *Cancer Res.*, **48**, 5713-5716.

MCGUIRE WP, HOSKINS WJ, BRADY MF, KUGERA PR, PARTRIDGE EE, LOOKKY P, FARSON DLC AND DAVIDSON M. (1993). A phase III trial comparing cisplatin/cytoxan and cisplatin/taxol in advanced ovarian cancer. *Proc. Am. Soc. Clin. Oncol.*, **12**, A808.

OZOLS RF. (1985). The case for combination chemotherapy in the treatment of advanced ovarian cancer. *J. Clin. Oncol.*, **3**, 1445-1447.

ROGAN AM, HAMILTON TC, YOUNG RC, KLECKER RW AND OZOLS RF. Reversal of adriamycin resistance by verapamil in human ovarian cancer. *Science*, **224**, 994-999.

ROWINSKY EK, GILBERT MR, MCGUIRE WP, NOE DA, GROCHON LB, FORASTIERE AA, ETTINGER DS, LUBEJKO BG, CLARK B, SARTORIUS SE, CORNBLATH DR, HENDRICKS CB AND DONEHOWER RC. (1991) Sequences of paclitaxel and cisplatin: a phase I and pharmacologic study. *J. Clin. Oncol.*, **9**, 1692-1703.

SKEHAN P, STORENG R, SCUDIERO D, MONKS A, MCMAHON J, VISTICA D, WARREN JT, BOKESCH H, KENNEY S AND BOYD MR. (1990). New colorimetric cytotoxicity assay for anticancer drug screening. *J. Natl Cancer Inst.*, **82**, 1107-1112.

THIGPEN JT, BLESSING JA, BALL H, HUMMELS J AND BARRETT RJ. (1994). Phase II trial of paclitaxel in patients with progressive ovarian carcinoma after platinum based chemotherapy: A gynecologic oncology group study. *J. Clin. Oncol.*, **12**, 1748-1753.

VANHOEFER U, HARSTRICK A, WILKE H, SCHLEUCHER N, WALLES H, SCHRÖDER J AND SEEBER S. (1995). Schedule-dependent antagonism of paclitaxel in human gastric and ovarian carcinoma cell lines in vitro. *Eur. J. Cancer*, **31A**(1), 92-97.